# Supplementary material for: Discovery of TDP-43 aggregation inhibitors via a hybrid machine learning framework
Source: bioRxiv. 2026 Feb 13:2026.02.12.705375. Preprint. [Version 1] doi: 10.64898/2026.02.12.705375 (PMC12918951; doi:10.64898/2026.02.12.705375)
Supplement: Supplement 1 [file media-1.pdf]

Discovery of TDP-43 aggregation inhibitors *via* a hybrid machine learning framework

Sofia Kapsiani[1], Sulay Vora[1], Ana Fernandez-Villegas[1], Clemens F. Kaminski[1], Nino F. Läubli[1*], Gabriele S. Kaminski Schierle[1,*]

[1] Department of Chemical Engineering and Biotechnology, University of Cambridge, Cambridge, CB3 0AS, UK

* Corresponding authors: nl431@cam.ac.uk & gsk20@cam.ac.uk

Contents

Supplementary Table 1. Performance metrics on 5-fold nested cross-validation for models trained using individual and combined feature types....2

Supplementary Table 2. Monte Carlo Tree Search-identified graph-based rationales associated with increased predicted for test set compounds correctly predicted as active....3

Supplementary Figure 1. Top performing features (n= 30) of the XGBoost model trained using GNN embeddings, RDKit chemical descriptions, and ChEMBL targets, identified using SHAP analysis....4

Supplementary Figure 2. Cell viability assessed by MTS assay following treatment with increasing concentrations of (a) berberrubine and (b) PE859 in HEK cells....5

Supplementary Figure 3. Treatment with candidate compounds significantly increases travel speed of hTDP-43 worms....6

Supplementary Table 1. Performance metrics on 5-fold nested cross-validation for models trained using individual and combined feature types. Random shuffling of target values (Y shuffle) served as a control to verify that performance was not attributable to information leakage.

Features	Classifier	ROC-AUC	MCC	F1-score	Balanced Accuracy	Precision
GNN embeddings + chemical descriptors + biological targets	XGBoost	0.84 ± 0.03	**0.33 ± 0.04**	**0.30 ± 0.06**	**0.59 ± 0.02**	0.73 ± 0.06
ECFPs (2048) chemical descriptors + biological targets	XGBoost	**0.87 ± 0.02**	0.30 ± 0.07	0.22 ± 0.08	0.56 ± 0.03	**0.84 ± 0.12**
chemical descriptors + biological targets	XGBoost	**0.87 ± 0.01**	0.28 ± 0.07	0.22 ± 0.11	0.57 ± 0.03	0.78 ± 0.16
biological targets	XGBoost	0.85 ± 0.03	0.21 ± 0.12	0.18 ± 0.13	0.55 ± 0.04	0.55 ± 0.17
chemical descriptors	XGBoost	0.74 ± 0.05	0.07 ± 0.10	0.03 ± 0.03	0.51 ± 0.01	0.50 ± 0.50
GNN network	MLP	0.70 ± 0.05	0.06 ± 0.08	0.03 ± 0.04	0.51 ± 0.01	0.30 ± 0.45
ECFPs (2048)	XGBoost	0.68 ± 0.04	0.10 ± 0.1	0.12 ± 0.08	0.53 ± 0.03	0.28 ± 0.17
random Y shuffle (GNN embeddings + chemical descriptors + biological targets)	XGBoost	0.50 ± 0.05	-0.01 ± 0.01	0.00 ± 0.00	0.50 ± 0.00	0.00 ± 0.00
GNN embeddings + chemical descriptors + biological targets	Random Forest	0.81 ± 0.04	0.17 ± 0.09	0.14 ± 0.07	0.54 ± 0.02	0.54 ± 0.23

Supplementary Table 2. Monte Carlo Tree Search-identified graph-based rationales associated with increased predicted for test set compounds correctly predicted as active. Rationales are highlighted in blue.

MLS003120626 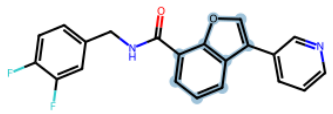	4-Chloro-7-methoxy-5H-pyrimido[5,4-b]indole 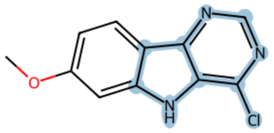	(2z)-N-(2,5-Dichlorophenyl)-2-(Hydroxyimino)-3,4-Dihydro-2h-1-Benzopyran-3-Carboxamide 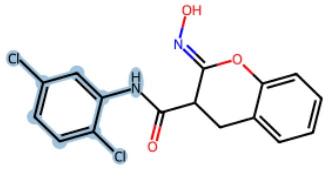
SMR001277452 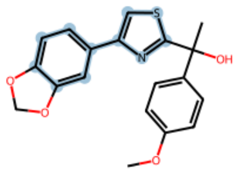	MLS003120491 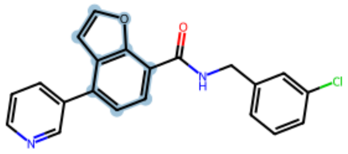	N-[2-(4-methoxyphenyl)quinolin-4-yl]-N',N'-dimethylpropane-1,3-diamine 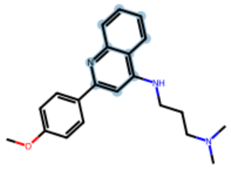
MLS003120620 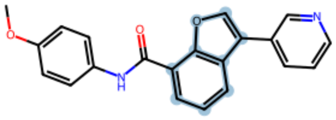	3-bromo-N-(2,3-dihydro-1,4-benzodioxin-6-ylcarbamothioyl)benzamide 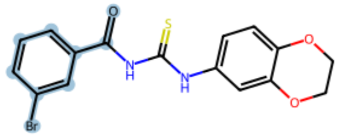	N-(1,3-benzothiazol-2-yl)-2-thiophen-2-ylacetamide 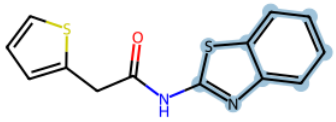
MLS001060488 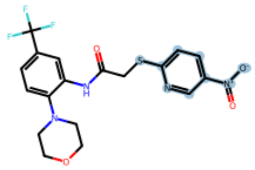	338748-09-7 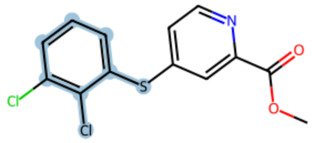	SMR000005287 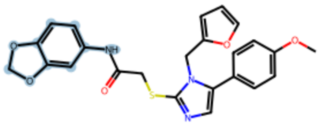

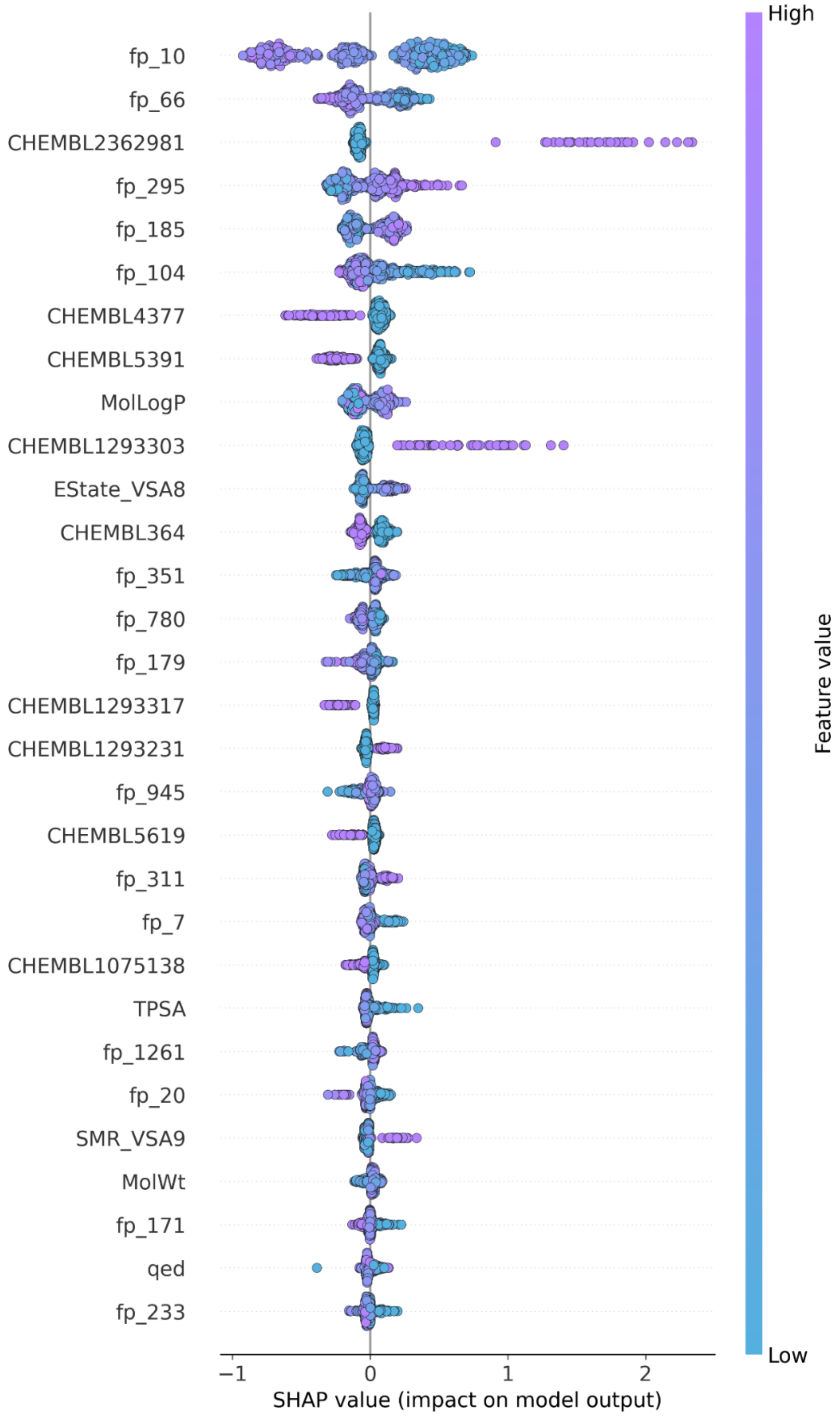

Supplementary Figure 1. Top performing features (n= 30) of the XGBoost model trained using GNN embeddings, RDKit chemical descriptions, and ChEMBL targets, identified using SHAP analysis. Half of the top-ranked descriptors correspond to GNN embeddings denoted by fp_. Points show individual compounds coloured by the corresponding SHAP value for that feature, where purple indicates a high SHAP value and blue represents a low one.

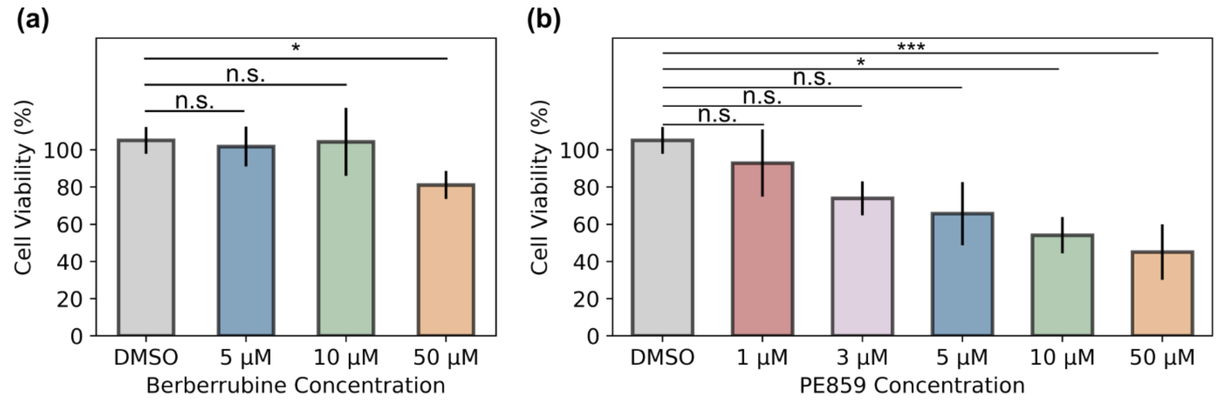

Supplementary Figure 2. Cell viability assessed by MTS assay following treatment with increasing concentrations of (a) berberrubine and (b) PE859 in HEK cells. Data were acquired from four biological replicates. Statistical comparisons were performed using one-way ANOVA with Tukey's HSD (berberrubine; normal distribution) or Kruskal–Wallis with Bonferroni-corrected Dunn's test (PE859; non-normal distribution). *$p < 0.05$, **$p < 0.01$, ***$p < 0.001$; n.s., non-significant.

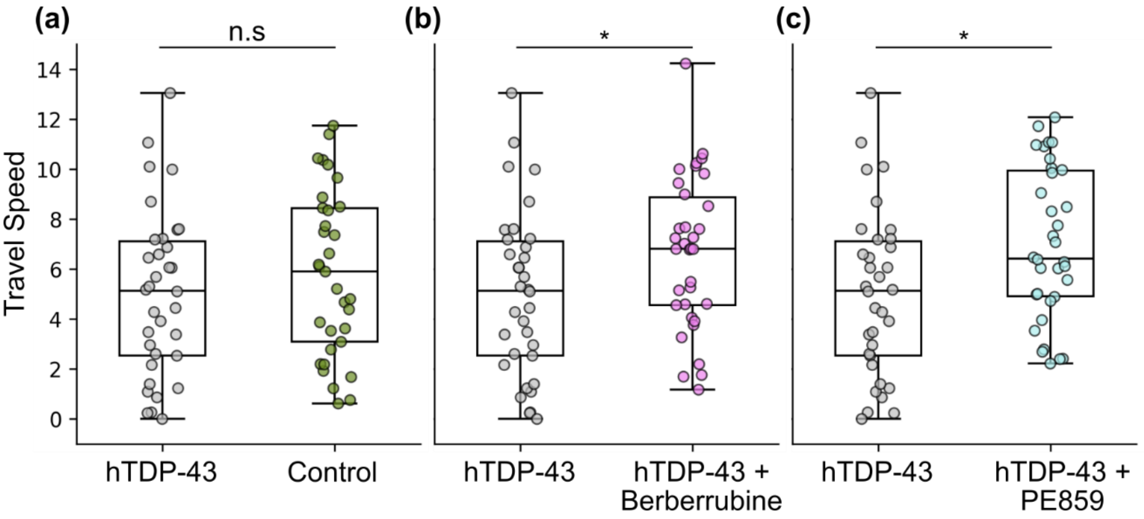

Supplementary Figure 3. Treatment with candidate compounds significantly increases travel speed of hTDP-43 worms. (a) Control worms display modestly higher locomotion speed compared to hTDP-43 worms, though this difference is not significant. Treatment with (b) berberrubine and (c) PE859 both significantly increase travel speed in worms expressing hTDP-43 pan-neuronally compared to untreated hTDP-43 worms. Statistical analysis was performed using Student's t-test, with *$p < 0.05$ and n.s. denoting not significant.
